# Revisional Adjustable Gastric Band in Roux-en-Y Gastric Bypass—Is It Worth It?

**DOI:** 10.1007/s11605-021-05045-7

**Published:** 2021-06-07

**Authors:** Ioannis I. Lazaridis, Marko Kraljević, Julian Süsstrunk, Thomas Köstler, Urs Zingg, Tarik Delko

**Affiliations:** 1 Clarunis, Department of Visceral Surgery, University Centre for Gastrointestinal and Liver Diseases, St. Clara Hospital and University Hospital Basel, 4002 Basel, Switzerland; 2 Obesity & Bariatric Surgery Centre, Department of Surgery, Limmattal Hospital, 8952 Zurich-Schlieren, Switzerland

**Keywords:** Bariatric surgery, Laparoscopic adjustable gastric banding, Roux-en-Y gastric bypass, Weight regain

## Abstract

**Purpose:**

A subset of patients undergoing Roux-en-Y gastric bypass (RYGB) presents with either insufficient weight loss or weight regain. Data on the revisional restrictive options including laparoscopic adjustable gastric band (LAGB) is scarce. This study analyzes the mid-term efficacy and safety of LAGB as a revisional procedure after RYGB.

**Methods:**

Data of all patients with revisional LAGB after primary RYGB between January 2011 and May 2019 were retrospectively reviewed. Outcomes included assessment of weight changes, resolution of comorbidities, and early and late complications during the study period.

**Results:**

Twenty patients were included. The median Body Mass Index (BMI) before revisional LAGB was 34.8 (interquartile range [IQR] 31.9–38.1) kg/m^2^. After a median follow-up of 33.5 (IQR 19.5–76.5) months, the median BMI was 28.7 (IQR 26.1–32.2) kg/m^2^. The median additional Excess Weight Loss (EWL) was 37.6% (IQR 23–44.4), leading to a median total EWL of 79.5% (IQR 54.4–94.6). BMI and EWL post-LAGB improved significantly compared to BMI and EWL pre-LAGB (p<0.001 and p<0.001, respectively). Obstructive sleep apnea syndrome resolved 6 months after LAGB in one patient. Three band deflations occurred during the follow-up. Six patients underwent band removal after a median time of 19 (IQR 15.8–26) months. Overall, thirteen patients underwent a reoperation. There was no loss of follow-up until 5 years. After that, two patients were lost to follow-up.

**Conclusion:**

LAGB may be a salvage option after failed RYGB. However, the high rate of revisions after secondary LAGB needs to be taken into consideration.

## Introduction

Roux-en-Y gastric bypass (RYGB) is the most effective treatment for obesity with durable weight loss and long-term improvement of comorbidities, such as type 2 diabetes mellitus (T2D), hypertension, and dyslipidemia.^1^ This efficacy has been well established in the majority of patients undergoing RYGB.^2^ However, a subset of patients presents with either insufficient weight loss, defined as an Excess Weight Loss (EWL) of less than 50% during follow-up or weight regain, defined as gain of at least 15% of the lowest post-operative weight.^3^,^4^ The failure rate varies among studies between 23 and 41%^5^–^7^ and is higher in super obese patients with a Body Mass Index (BMI) over > 50kg/m^2^.^8^

Patients with insufficient weight loss or weight regain as well as persistence or recurrence of comorbidities after RYGB may need further treatment. Unsatisfactory outcomes after RYGB may be caused by an unfavorable lifestyle, metabolic dysfunction, mental disorders, or rarely by anatomical failures. The latter include the dilatation of the gastric pouch or the gastroenterostomy, respectively, or the development of a gastrogastric fistula between the pouch and the gastric remnant.^9^,^10^ Because of the multifactorial etiology of failure after RYGB, several treatment options have been proposed, including behavioral modifications, and endoscopic or surgical revisional techniques. The latter are classified into malabsorptive and restrictive revisional procedures. Malabsorptive techniques include conversion to a distal bypass such as the very-very-long-limb Roux-en-Y gastric bypass (VVLL-RYGB) or a RYGB with a long biliopancreatic limb (BPL RYGB) as well as the classic biliopancreatic diversion with duodenal switch (BPD/DS). The restrictive options consist of a laparoscopic resizing of the gastric pouch or the implantation of a non-adjustable or adjustable gastric band.^11^

At present, data on the revisional restrictive options is scarce. Most studies investigating laparoscopic adjustable gastric band (LAGB) secondary to RYGB report only short-term outcomes. The aim of this study is to analyze the mid-term efficacy and safety of the LAGB as a revisional procedure after index RYGB.

## Methods

### Design and Subjects

Data of all consecutive patients with implantation of an adjustable gastric band after primary RYGB between January 2011 and May 2019 at Limmattal Hospital in Zurich-Schlieren, the second largest bariatric center in Switzerland, has been included in this study. If a band removal was performed, only the patients’ data until the band removal have been analyzed. Exclusion criterion was the concomitant use of other revisional techniques such as lengthening of either the Roux or biliopancreatic limbs. Baseline characteristics, early and late morbidity during follow-up, and changes in EWL and Body Mass Index (BMI) were registered. The local Ethics Committee approved the database and its analysis.

### Pre-revisional Assessment and Surgical Technique

All procedures were performed laparoscopically by a single experienced bariatric surgeon (TK). The primary bariatric procedure was either standard proximal Roux-en-Y gastric bypass (PRYGB) or VVLL-RYGB, as described by the Mayo Clinic, Rochester, MN.^12^ For PRYGB, a biliopancreatic limb (BPL) of 50–60 cm and an alimentary limb (AL) of 150 cm were formed. For VVLL-RYGB, BPL length was 50–60cm and common channel (CC) length was between 100 and 150 cm. Because of inadequate weight loss, conversion of PRYGB into VVLL-RYGB or BPL RYGB had previously been performed in some patients before secondary LAGB. For BPL RYGB, BPL length was 250–300cm and CC length was 100–150cm. Gastrojejunostomy was performed using a Premium Plus CEEA^TM^ 25-mm circular stapler (Covidien, Dublin, Republic of Ireland). Entero-enterostomy was performed using the Echelon Flex™ Powered Endopath® Stapler (60mm, white; Ethicon, Somerville, NJ, USA), closing the enteric defect with a running absorbable PDS® (polydioxanone) 3-0 suture (Ethicon).

All patients that demonstrated insufficient weight loss or weight regain were assessed by a multidisciplinary care team (endocrinologist, dietitian, psychiatrist, and bariatric surgeon) according to the guidelines of the Swiss Society for the Study of Morbid Obesity and Metabolic Disorders (SMOB). Patients underwent a comprehensive evaluation for behavioral failures such as binge eating, emotional eating, and loss-of-control over eating. The initial step of treatment included lifestyle, diet modifications, and behavioral interventions. Patients with persistent failure were then evaluated for revisional surgery. Eligibility criteria were either inadequate weight loss (EWL < 50% and/or BMI > 35 kg/m^2^) or weight regain (15% of the lowest post-operative weight) 24 months after the last bariatric procedure. Anatomical failures such as dilatation of the gastric pouch or the gastrojejunostomy and gastrogastric fistula were excluded. The anatomy of the pouch and the gastrojejunostomy was evaluated by upper endoscopy, contrast studies, or contrast computed tomography (CT). Corresponding to previous studies, a pouch was considered enlarged if >6cm in length and >5 cm in width; a gastrojejunostomy was classified as dilated if > 2 cm in diameter.^13^,^14^

In all patients, the MiniMizer Extra© adjustable gastric band (Bariatric Solutions, Stein am Rhein, Switzerland) was used. The band was inserted around the gastroesophageal junction with the pars flaccida technique. The band was left empty without volume adjustment for at least 6 weeks post-operatively.

### Post-operative Management

Follow-up was obtained by the bariatric surgeon, dietitian, and endocrinologist at 6 weeks and every 6 months thereafter on an outpatient basis over the entire study period. Reviews during visits included weight change, subjective feeling of band restriction, stool frequency, laboratory tests, change of comorbidities, and assessment of post-operative complications. Band adjustments were performed based on the clinical assessment of the patient’s subjective feeling of food intake restriction and the presence of obstructive symptoms such as vomiting, dysphagia, and/or reflux. The filling volume during the first adjustment was between 1.5 and 3.5 ml. All further adjustments were made in 0.5 ml steps under fluoroscopic guidance. Delayed contrast passage across the band during the post-adjustment contrast studies with presence of mild-narrowing of the gastric lumen and absence of obstructive symptoms after intake of 1dl of water was perceived as a satisfactory result.

### Outcome Measures

Primary outcome was the assessment of weight changes during the study period, including before the index and the revisional bariatric procedures. Secondary outcomes included the incidence of comorbidities and early and late complications according to the Clavien/Dindo classification. The weight outcomes were recorded as follows: BMI, ΔBMI (initial BMI – post-operative BMI), % Total Weight Loss (%TWL) defined as [(Initial Weight) – (Post-operative Weight)] / (Initial Weight) × 100 and %EWL defined as [(Initial Weight) – (Post-operative Weight)] / [(Initial Weight) – (Ideal Weight)] × 100. Ideal weight was defined by the weight corresponding to a BMI of 25 kg/m^2^. Comorbidities evaluated pre- and post-operatively included T2D, arterial hypertension, abnormal lipid profile, gastroesophageal reflux disease (GERD), and obstructive sleep apnea syndrome (OSAS). Definitions of comorbidities were as follows: arterial hypertension (systolic blood pressure > 140 mmHg, diastolic blood pressure > 90 mmHg with/without use of antihypertensive medication), T2D (Glycosylated Hemoglobin > 6.5% with/without the use of antidiabetic medication), hyperlipidemia (elevated cholesterol and/or triglycerides), GERD (esophagitis ≥ grade B according to the Los Angeles classification), and OSAS (at least five events of apnea or hypopnea per hour diagnosed in an overnight polysomnogram).

### Statistical Analysis

Continuous data is presented as mean ± standard deviation (SD) or median with interquartile range (IQR) as appropriate. Comparison of BMI and %EWL changes was performed using the Wilcoxon rank test. Statistical significance was defined by p < 0.05. All data analysis was performed using R version 3.5.3.^15^

## Results

### Pre-operative Descriptives

Between January 2011 and May 2019, twenty-one patients (n=21) underwent additional LAGB after RYGB. One patient underwent concomitant conversion of PRYGB into VVLL-RYGB and was not included in the study, leaving twenty eligible patients (n=20, 4 males). Index operations before additional gastric banding were as follows: seven patients with PRYGB, ten patients with VVLL-RYGB, one patient with conversion from PRYGB to VVLL-RYGB, one patient with conversion from PRYGB to BPL RYGB, and one patient with conversion from VVLL-RYGB to BPL RYGB. The median BMI before the initial bariatric operation was 42.5 (41.3–45.4) kg/m^2^. The median time from the last bariatric procedure until secondary LAGB was 51.5 (31–85.3) months. There was no loss of follow-up until 5 years. After that, 2 patients (10%) were lost to follow-up.

### Comorbidities

All patients had a complete assessment of their comorbidities at the last follow-up. Prior to revisional surgery, two patients (10%) had arterial hypertension, which did not resolve at the time of the last follow-up. One patient (5%) had OSAS, which resolved after 6 months.

### Perioperative Data

Fifteen patients underwent revisional LAGB because of weight regain and five patients because of insufficient weight loss. The median BMI before revisional LAGB was 34.8 (31.9–38.1) kg/m^2^. The mean age at the time of LAGB was 46.6 ± 10.8 years. The median operative time was 119 (90–133) min. All operations were performed laparoscopically. The median length of hospital stay was 3 (3–4) days.

### Weight Loss and BMI Changes

BMI and EWL changes for each individual patient during the study period are presented in Table 1. After a median follow-up of 33.5 (19.5–76.5) months between revisional LAGB and last follow-up, the median BMI was 28.7 (26.1–32.2) kg/m^2^, leading to a median ΔBMI of 6.1 (3.6–8) kg/m^2^. The median additional EWL was 37.6% (23–44.4), leading to a median total EWL of 79.5% (54.4–94.6). BMI and EWL post-LAGB improved significantly compared to BMI and EWL pre-LAGB (p<0.001 and p<0.001, respectively). BMI, ΔBMI, EWL, and TWL changes over time including number of available/eligible patients at every time point are shown in Table 2.

**Table 1:** Patients’ demographic characteristics and weight loss outcomes

	Gender	Initial BMI (kg/m^2^)	Last bariatric operation before LAGB	Pre-revisional BMI (kg/m^2^)	FU (months)	BMI at last FU (kg/m^2^)	Band status
1	Female	41.4	VVLL-RYGB	30.9	91	24.5	In situ
2	Female	43.7	VVLL-RYGB	37.5	72	27.1	In situ
3	Female	61.9	VVLL-RYGB	40.2	87	42.7	In situ
4	Female	42.1	VVLL-RYGB	38.4	59	28.0	In situ
5	Female	48.4	PRYGB	39.4	15	35.9	Removed
6	Female	42.4	VVLL-RYGB	33.8	84	26.5	In situ
7	Female	43.6	VVLL-RYGB	30.8	81	22.7	In situ
8	Female	40.4	VVLL-RYGB	37.8	75	32.0	In situ
9	Female	42.3	PRYGB	32.1	81	33.0	Deflated
10	Female	41.0	VVLL-RYGB	33.5	60	24.7	Deflated
11	Female	50.7	PRYGB	34.9	27	37.6	In situ
12	Male	39.9	PRYGB	31.2	20	23.7	Removed
13	Female	36.5	BPL RYGB	28.8	25	23.4	In situ
14	Male	59.5	BPL RYGB	40.7	38	27.7	Removed
15	Female	45.4	VVLL-RYGB	34.6	29	29.8	In situ
16	Male	42.9	VVLL-RYGB	33.9	9	29.4	Removed
17	Female	45.5	VVLL-RYGB	39.8	18	31.9	Removed
18	Female	42.0	PRYGB	36.4	28	32.9	Removed
19	Male	37.6	PRYGB	30.3	12	26.7	In situ
20	Female	42.7	PRYGB	38.0	18	31.7	In situ

*BMI* Body Mass Index, *LAGB* laparoscopic adjustable gastric banding, *FU* follow-up, *PRYGB* proximal Roux-en-Y gastric bypass, *VVLL-RYGB* very-very-long-limb Roux-en-Y gastric bypass, *BPL RYGB* Roux-en-Y gastric bypass with long biliopancreatic limb

**Table 2:** BMI, ΔBMI, %EWL, and %TWL changes over the study period

	Before index operation	Nadir	Pre-LAGB	FU 1 year	FU 2 years	FU 3 years	FU 4 years	FU 5 years	FU 6 years	FU 7 years	FU 8 years
Available subjects (n)	20	20	20	20	18	12	9	9	7	5	1
Eligible subjects (n)	20	20	20	20	18	12	9	9	8	6	1
BMI (kg/m^2^)	42.5 (41.3–45.4)	29.1 (27.1–35.1)	34.8 (31.9–38.1)	31.6 (27.9–33.8)	31 (24.9–32.7)	27.7 (26.1–33)	26.9 (23.6–27.4)	24.7 (23.6–28)	26.5 (24.1–27.1)	26.5 (24.5–33)	24.8
ΔBMI (kg/m^2^)	-	13.4 (9.6–17.1)	8.7 (6.1–10–6)	13.0 (10.6–15.2)	14.0 (11.4–18.2)	16.8 (11.8–19.8)	17.4 (15.2–20)	16.3 (14.1–18.8)	16.6 (15.9–17.3)	16.9 (15.9–19.2)	16.6
%ΕWL	-	74.4 (54.9–83.0)	51.7 (33.3–58.9)	72.4 (54.5–83.6)	69.2 (55.5–100)	91.5 (54–94.5)	86.3 (77.2–109)	102 (82.3–109)	89.6 (88.6–106)	97.9 (53.6–104)	102.8
%TWL	-	31.1 (24.9–37.2)	20.8 (14.1–24.5)	30.7 (24.1–35.1)	31.8 (25.8–40.2)	36.2 (27.1–43)	35.5 (32.4–42.6)	39.8 (33.4–46.6)	37.9 (36.8–42.5)	40.2 (31–40.4)	41

Data are expressed in median values (interquartile range; Q1 first quartile; Q3 third quartile)

*BMI* Body Mass Index, *ΔBMI* initial BMI – post-operative BMI, *%EWL* Excess Weight Loss (%), *%TWL* Total Weight Loss (%), *LAGB* laparoscopic adjustable gastric banding, *FU* follow-up

### Morbidity and Mortality

No intraoperative or early morbidity occurred. One 30-day hospital readmission (5%) occurred because of epigastric pain, with no specific pathology found.

Two cases of de novo GERD (10%) were diagnosed. One patient had erosive esophagitis Los Angeles Classification Grade B and one patient developed Barrett’s esophagus, respectively. In the former patient, the band was deflated, resulting in complete resolution of symptoms. The latter patient underwent revision into BPL RYGB because of concomitant weight rebound. Two additional patients underwent band deflation. The first demonstrated dysphagia and malnutrition, while the second patient complained of dysphagia, vitamin D, and iron deficiency. The latter underwent upper GI endoscopy, which revealed an anastomotic ulcer. The symptoms resolved after band deflation and treatment with proton-pump inhibitors.

Six patients (30%) underwent band removal after a median time of 19 (15.8–26) months; one patient because of slippage and the other five because of intolerable dysphagia. Three patients (15%) needed port replacement because of disconnection. One patient (5%) underwent band replacement due to defective band system. One port refixation occurred because of torsion of the alimentary limb around the tube. The median (IQR) number of band adjustments was 3 (1–5). Overall, thirteen patients (65%) underwent a reoperation during the follow-up. Table 3 shows a summary of the post-operative complications according to the Clavien-Dindo classification.

**Table 3:** Post-operative complications during follow-up

Grade (Clavien-Dindo classification)	Complication type	*n (%)*
IIIa	Band deflation	3 (15)
IIIa	Anastomotic ulcer	1 (5)
IIIb	Band replacement	1 (5)
IIIb	Band removal	6 (30)
IIIb	Port-associated complications	4 (20)
IIIb	Tube-associated complications	1 (5)
IIIb	Revision to BPL RYGB (insufficient weight loss)	1 (5)

## Discussion

The main finding of this study is the significant additional weight loss achieved by the placement of an adjustable gastric band in patients with insufficient weight loss or weight regain after RYGB. The banding of the gastric bypass leads to a median additional EWL of 37.6% and to a further median BMI reduction of 6.1kg/m^2^ after a follow-up of 33.5 months. These outcomes are consistent with the weight loss outcomes reported in the literature.^16^–^20^ The data of our study is based on a longer follow-up period than the majority of the previous studies. Overall, only 94 patients’ mostly short-term outcomes have been published until 2012.^21^ To our knowledge, four studies have been published since then, reporting the results of a total of 297 patients. The mean/median follow-up of these studies varies among 14 months and 2.48 years and in the series of Liu et al. 20 out of 86 patients were followed-up at 5 years.^16^–^19^

Laparoscopic RYGB is one of the most effective bariatric procedures with durable weight loss outcomes, acceptable morbidity, and low mortality.^22^–^24^ However, the issue of insufficient weight loss or weight regain after RYGB is well recognized.^25^ Anatomical, behavioral, and hormonal factors contribute to the unfavorable outcomes after RYGB.^10^,^26^ If medical and conservative multidisciplinary treatment fails to manage weight regain, surgical revision options may lead to further weight loss and control of comorbidities. Strict criteria to choose the most appropriate revisional procedure for the individual patient are still lacking. The alteration of the length of the alimentary or the biliopancreatic limb shows successful additional weight loss outcomes in well-selected and highly compliant patients. Still, these patients are at risk of developing severe nutritional deficiencies with the need for parenteral nutrition or reversal to standard RYGB.^27^–^29^ Pouch resizing for patients with weight regain and gastric pouch dilatation is an accepted treatment, but the results of this procedure have not been widely studied in the long term.^30^–^32^ The reduction of the gastrojejunal stoma diameter for patients with secondary stoma dilatation does not seem to offer a major weight loss effect in the long term, probably because of reoccurring dilatation of the gastrojejunal anastomosis.^33^ Banding of the gastric pouch has been described in the primary and the revisional setting. As a primary measure during RYGB, described by Fobi et al. in 1989, a silastic ring band is placed along the lower third of the pouch.^34^ Banded RYGB is suggested to maintain long-term weight loss with low complication rates.^35^,^36^ Additional secondary placement of adjustable or non-adjustable bands to manage failure of RYGB has been described in numerous studies and demonstrates promising short-term outcomes.^11^,^16^–^18^,^21^,^37^–^40^

Due to the lack of a standardized treatment algorithm for the surgical management of failed RYGB, the choice of the revisional procedure should be made in a multidisciplinary and patient-tailored approach. In our clinical setting, revisional LAGB was offered to patients with a moderate weight regain or insufficient weight loss, respectively. The median BMI at the time of revisional LAGB was 34.8 kg/m^2^ and only two patients had a pre-revisional BMI higher than 40kg/m^2^. As previously reported, revisional malabsorptive procedures were chosen for patients with a higher mean pre-revisional BMI of 41.7 ± 4.4 kg/m^2^.^41^ Another selection criterion for revisional LAGB was the pouch anatomy; after thorough pre-operative endoscopic and radiological examination, secondary LAGB was performed only in patients presenting with a normal pouch size. In case of a dilated pouch, pouch resizing was performed.

Most patients with weight regain reported of behavioral or diet-related factors, including increased caloric intake over time, big eating, and binge eating. The addition of a restriction via an adjustable band represented an effective approach to manage poor diet quality after nutritional counseling had failed.

In this series, revisional LAGB was not only performed after failed primary PRYGB, but also in ten patients after primary VVLL-RYGB. In three cases, a revisional malabsorptive procedure (BPL RYGB or VVLL RYGB) had been previously performed after failure of the primary PRYGB procedure. In these thirteen patients with prior malabsorptive procedures, the additional band placement contributed to an additional weight loss or the prevention of weight regain. This indicates that the LAGB may also be a salvage option in patients that have already undergone a malabsorptive treatment.

Revisional bariatric surgery after failed RYGB is technically demanding and associated with a significant morbidity, such as anastomotic leaks; superficial, deep, or organ space surgical site infections; and high reoperation rates.^42^,^43^ Our study shows that LAGB after RYGB is a safe and feasible procedure with a low early post-operative morbidity. An important advantage of secondary LAGB is its relative technical simplicity and the absence of a new gastrointestinal anastomosis or any stapling, thus avoiding the risk of a leak. However, the reoperation rate in this series in the long term was high with 65%. The high rate of reoperations due to band-related problems raises concerns about a widespread use of this technique. These reoperation rates are higher than those mentioned in other studies of secondary LAGB, probably because of the longer follow-up and the lower rates of loss of follow-up. The post-operative complications in our series included dysphagia, GERD, and nutritional deficiencies and could be managed and reversed through band deflation or band removal. Port-associated complications were managed with port replacement and refixation, which are technically simple procedures with low operative risk, well tolerated by the patients. Still, an overall reoperation rate of 65% is unacceptable. Thus, the indication for a LAGB after failed RYGB type procedures must be taken with care and other options must be scrutinized first. However, conservative treatment often fails and malabsorptive revisional surgery may have its own severe consequences. The revisional LAGB is one of different options for the bariatric surgeon confronted with weight loss failure.

The number of the primary LAGB procedures progressively declined in recent years.^44^ In contrast to revisional LAGB after RYGB bypass, primary LAGB has been associated with poor long-term weight loss outcomes. The long-term morbidity such as dysphagia and reflux, however, is similar and requires frequent revision in both situations. The practice of band adjustments to manage dysphagia and food intolerance has been criticized because of the difficulty in fine-tuning.^45^ High rates of band revision and removal after primary implantation, up to 59.4%, have been reported in the literature.^46^ In contrast to primary LAGB, the reported removal rates of secondary LAGB after failed RYGB are up to 21%, but these rates refer to smaller sample sizes and to shorter follow-up periods.^16^–^20^ In our study, six (30%) patients required band removal after a median follow-up of 33.5 months. The lower band removal rates reported in the previous studies may be explained by their higher rates of loss of follow-up (between 14 and 35.3% after 12 months).^16^–^18^,^20^ In contrast, no patients were lost to follow-up until 5 years in our series. Although the existing data are insufficient to draw safe conclusions, the lower removal rate of secondary LAGB could be explained by the anti-reflux effect of the RYGB procedure. The separation of the major part of the stomach from the gastroesophageal junction leads to a lower exposure to acid or bile, in case of non-acid reflux. Intolerable GERD is one of the major reasons for band removal. In addition, due to the small volume of the gastric pouch, the incidence of band slippage may be lower among patients receiving secondary LAGB after RYGB than the slippage incidence after primary LAGB.

The limitations of this study include its retrospective, observational design. Furthermore, we only observed a highly selected cohort of patients. Due to the strict selection criteria in our multidisciplinary team setting, only a small number of patients qualified to receive revisional RYGB banding. However, due to the scarcity of long-term data on revisional banding after RYGB, we believe that this study with a median follow-up of 33.5 months and without loss of follow-up until 5 years provides additional insight into this topic. In addition, our study reports the outcome of revisional banding in different RYGB variations such as PRYGB, VVLL-RYGB, and BPL RYGB. Further research focusing on the comparison of the existing revisional techniques may aid in directing future guidelines regarding revisional surgery after failed RYGB.

## Conclusion

LAGB may be a salvage option after failed RYGB. The increase of restriction by the placement of an adjustable band can be considered as a therapeutic option for patients with insufficient weight loss or weight regain after RYGB. However, the high rate of revisions after secondary LAGB needs to be taken into consideration. Proper patient selection should be made through a multidisciplinary pre-operative evaluation.

## References

[1] Cohen R, Le Roux CW, Junqueira S, Ribeiro RA, Luque A (2017). Roux-En-Y Gastric Bypass in Type 2 Diabetes Patients with Mild Obesity: a Systematic Review and Meta-analysis. Obes Surg..

[2] Chang SH, Stoll CRT, Song J, Varela JE, Eagon CJ, Colditz GA. The effectiveness and risks of bariatric surgery an updated systematic review and meta-analysis, 2003-2012. *JAMA Surg*. 2014. 10.1001/jamasurg.2013.3654 10.1001/jamasurg.2013.3654 PMC3962512 24352617

[3] Amundsen T, Strømmen M, Martins C. Suboptimal Weight Loss and Weight Regain after Gastric Bypass Surgery—Postoperative Status of Energy Intake, Eating Behavior, Physical Activity, and Psychometrics. *Obes Surg*. 2017. 10.1007/s11695-016-2475-7 10.1007/s11695-016-2475-7 PMC5403843 27914028

[4] Odom J, Zalesin KC, Washington TL, et al. Behavioral predictors of weight regain after bariatric surgery. *Obes Surg*. 2010. 10.1007/s11695-009-9895-6 10.1007/s11695-009-9895-6 19554382

[5] Monaco-Ferreira DV, Leandro-Merhi VA. Weight Regain 10 Years After Roux-en-Y Gastric Bypass. *Obes Surg*. 2017. 10.1007/s11695-016-2426-3 10.1007/s11695-016-2426-3 27798793

[6] Nicoletti CF, de Oliveira BAP, de Pinhel MAS, et al. Influence of Excess Weight Loss and Weight Regain on Biochemical Indicators During a 4-Year Follow-up After Roux-en-Y Gastric Bypass. *Obes Surg*. 2015. 10.1007/s11695-014-1349-0 10.1007/s11695-014-1349-0 24996801

[7] Cooper TC, Simmons EB, Webb K, Burns JL, Kushner RF. Trends in Weight Regain Following Roux-en-Y Gastric Bypass (RYGB) Bariatric Surgery. *Obes Surg*. 2015. 10.1007/s11695-014-1560-z 10.1007/s11695-014-1560-z 25595383

[8] Christou N V., Look D, MacLean LD. Weight gain after short- and long-limb gastric bypass in patients followed for longer than 10 years. *Ann Surg*. 2006. 10.1097/01.sla.0000217592.04061.d5 10.1097/01.sla.0000217592.04061.d5 PMC1856611 17060766

[9] Campos GM, Rabl C, Mulligan K, et al. Factors associated with weight loss after gastric bypass. *Arch Surg*. 2008. 10.1001/archsurg.143.9.877 10.1001/archsurg.143.9.877 PMC2747804 18794426

[10] Maleckas A, Gudaitytė R, Petereit R, Venclauskas L, Veličkienė D. Weight regain after gastric bypass: etiology and treatment options. *Gland Surg*. 2016. 10.21037/gs.2016.12.02 10.21037/gs.2016.12.02 PMC5233838 28149808

[11] Tran DD, Nwokeabia ID, Purnell S (2016). Revision of Roux-En-Y Gastric Bypass for Weight Regain: a Systematic Review of Techniques and Outcomes. Obes Surg..

[12] Nelson WK, Fatima J, Houghton SG (2006). The malabsorptive very, very long limb Roux-en-Y gastric bypass for super obesity: Results in 257 patients. Surgery..

[13] Brethauer SA, Nfonsam V, Sherman V, Udomsawaengsup S, Schauer PR, Chand B (2006). Endoscopy and upper gastrointestinal contrast studies are complementary in evaluation of weight regain after bariatric surgery. Surg Obes Relat Dis..

[14] Yimcharoen P, Heneghan HM, Singh M (2011). Endoscopic findings and outcomes of revisional procedures for patients with weight recidivism after gastric bypass. Surg Endosc..

[15] R Core Team. R: A Langauge and environment for statistical computing. (Computer software). 2018.

[16] Schmidt HJ, Lee EW, Amianda EA, et al. Large series examining laparoscopic adjustable gastric banding as a salvage solution for failed gastric bypass. *Surg Obes Relat Dis*. 2018. 10.1016/j.soard.2018.09.003 10.1016/j.soard.2018.09.003 30309778

[17] Aminian A, Corcelles R, Daigle CR, Chand B, Brethauer SA, Schauer PR. Critical appraisal of salvage banding for weight loss failure after gastric bypass. *Surg Obes Relat Dis*. 11(3):607-611. 10.1016/j.soard.2014.11.014 10.1016/j.soard.2014.11.014 26093767

[18] Uittenbogaart M, Leclercq WK, Luijten AA, van Dielen FM (2017). Laparoscopic Adjustable Gastric Banding After Failed Roux-En-Y Gastric Bypass. Obes Surg..

[19] Liu S, Ren-Fielding CJ, Schwack B, Kurian M, Fielding GA (2018). Long-term results for gastric banding as salvage procedure for patients with weight loss failure after Roux-en-Y gastric bypass. Surg Obes Relat Dis..

[20] Irani K, Youn HA, Ren-Fielding CJ, Fielding GA, Kurian M (2011). Midterm results for gastric banding as salvage procedure for patients with weight loss failure after Roux-en-Y gastric bypass. Surg Obes Relat Dis..

[21] Vijgen GHEJ, Schouten R, Bouvy ND, Greve JWM (2012). Salvage banding for failed Roux-en-Y gastric bypass. Surg Obes Relat Dis..

[22] Gu L, Huang X, Li S (2020). A meta-analysis of the medium- and long-term effects of laparoscopic sleeve gastrectomy and laparoscopic Roux-en-Y gastric bypass. BMC Surg..

[23] Han Y, Jia Y, Wang H, Cao L, Zhao Y (2020). Comparative analysis of weight loss and resolution of comorbidities between laparoscopic sleeve gastrectomy and Roux-en-Y gastric bypass: A systematic review and meta-analysis based on 18 studies. Int J Surg..

[24] Kauppila JH, Santoni G, Tao W, et al. Reintervention or mortality within 90 days of bariatric surgery: population-based cohort study. *BJS (British J Surgery)*. 2020;n/a(n/a). 10.1002/bjs.11533 10.1002/bjs.11730 32497245

[25] Karmali S, Brar B, Shi X, Sharma AM, de Gara C, Birch DW (2013). Weight Recidivism Post-Bariatric Surgery: A Systematic Review. Obes Surg..

[26] King WC, Belle SH, Hinerman AS, Mitchell JE, Steffen KJ, Courcoulas AP. Patient Behaviors and Characteristics Related to Weight Regain After Roux-en-Y Gastric Bypass: A Multicenter Prospective Cohort Study. *Ann Surg*. 9000;Publish Ah. https://journals.lww.com/annalsofsurgery/Fulltext/9000/Patient_Behaviors_and_Characteristics_Related_to.95143.aspx. 10.1097/SLA.0000000000003281 PMC8725202 30950861

[27] Buchwald H, Oien DM (2017). Revision Roux-en-Y Gastric Bypass to Biliopancreatic Long-Limb Gastric Bypass for Inadequate Weight Response: Case Series and Analysis. Obes Surg..

[28] Ghiassi S, Higa K, Chang S (2018). Conversion of standard Roux-en-Y gastric bypass to distal bypass for weight loss failure and metabolic syndrome: 3-year follow-up and evolution of technique to reduce nutritional complications. Surg Obes Relat Dis..

[29] Rawlins ML, Teel D, Hedgcorth K, Maguire JP (2011). Revision of Roux-en-Y gastric bypass to distal bypass for failed weight loss. Surg Obes Relat Dis..

[30] Iannelli A, Schneck A-S, Hébuterne X, Gugenheim J. Gastric pouch resizing for Roux-en-Y gastric bypass failure in patients with a dilated pouch. *Surg Obes Relat Dis*. 9(2):260-267. 10.1016/j.soard.2012.05.003 10.1016/j.soard.2012.05.003 22695174

[31] Al-Bader I, Khoursheed M, Al Sharaf K, Mouzannar DA, Ashraf A, Fingerhut A (2015). Revisional Laparoscopic Gastric Pouch Resizing for Inadequate Weight Loss After Roux-en-Y Gastric Bypass. Obes Surg..

[32] Borbély Y, Winkler C, Kröll D, Nett P (2017). Pouch Reshaping for Significant Weight Regain after Roux-en-Y Gastric Bypass. Obes Surg..

[33] Parikh M, Heacock L, Gagner M (2011). Laparoscopic “Gastrojejunal Sleeve Reduction” as a Revision Procedure for Weight Loss Failure After Roux-En-Y Gastric Bypass. Obes Surg..

[34] Fobi M (2005). Banded gastric bypass: Combining two principles. Surg Obes Relat Dis..

[35] Lemmens L (2017). Banded Gastric Bypass: Better Long-Term Results? A Cohort Study with Minimum 5-Year Follow-Up. Obes Surg..

[36] Heneghan HM, Annaberdyev S, Eldar S, Rogula T, Brethauer S, Schauer P. Banded Roux-en-Y gastric bypass for the treatment of morbid obesity. *Surg Obes Relat Dis*. 10(2):210-216. 10.1016/j.soard.2013.10.016 10.1016/j.soard.2013.10.016 24462315

[37] Bessler M, Daud A, Inabnet WB, Schrope B, Davis D (2008). PL-57: Adjustable gastric banding as a revisional bariatric procedure after failed gastric bypass - intermediate results. Surg Obes Relat Dis..

[38] Dapri G, Cadière GB, Himpens J (2009). Laparoscopic Placement of Non-Adjustable Silicone Ring for Weight Regain After Roux-en-Y Gastric Bypass. Obes Surg..

[39] Chin PL, Ali M, Francis K, LePort PC (2009). Adjustable gastric band placed around gastric bypass pouch as revision operation for failed gastric bypass. Surg Obes Relat Dis..

[40] Boerboom A, Aarts E, Lange V (2020). Banding the Pouch with a Non-adjustable Ring as Revisional Procedure in Patients with Insufficient Results After Roux-en-Y Gastric Bypass: Short-term Outcomes of a Multicenter Cohort Study. Obes Surg..

[41] Kraljević M, Köstler T, Süsstrunk J (2020). Revisional Surgery for Insufficient Loss or Regain of Weight After Roux-en-Y Gastric Bypass: Biliopancreatic Limb Length Matters. Obes Surg..

[42] Himpens J, Coromina L, Verbrugghe A, Cadière G-B (2012). Outcomes of revisional procedures for insufficient weight loss or weight regain after Roux-en-Y gastric bypass. Obes Surg..

[43] Patel S, Szomstein S, Rosenthal RJ (2011). Reasons and outcomes of reoperative bariatric surgery for failed and complicated procedures (excluding adjustable gastric banding). Obes Surg..

[44] Angrisani L, Santonicola A, Iovino P, Formisano G, Buchwald H, Scopinaro N (2015). Bariatric Surgery Worldwide 2013. Obes Surg..

[45] Dargent J (2017). Laparoscopic Gastric Banding: Game Over?. Obes Surg..

[46] Froylich D, Abramovich-Segal T, Pascal G (2018). Long-Term (over 10 Years) Retrospective Follow-up of Laparoscopic Adjustable Gastric Banding. Obes Surg..

